# The Role of Leukotrienes as Potential Therapeutic Targets in Allergic Disorders

**DOI:** 10.3390/ijms20143580

**Published:** 2019-07-22

**Authors:** Airi Jo-Watanabe, Toshiaki Okuno, Takehiko Yokomizo

**Affiliations:** Department of Biochemistry, Juntendo University School of Medicine, Tokyo 113-8421, Japan

**Keywords:** leukotriene, LTB_4_, BLT1, cysteinyl leukotriene, allergic disease, asthma

## Abstract

Leukotrienes (LTs) are lipid mediators that play pivotal roles in acute and chronic inflammation and allergic diseases. They exert their biological effects by binding to specific G-protein-coupled receptors. Each LT receptor subtype exhibits unique functions and expression patterns. LTs play roles in various allergic diseases, including asthma (neutrophilic asthma and aspirin-sensitive asthma), allergic rhinitis, atopic dermatitis, allergic conjunctivitis, and anaphylaxis. This review summarizes the biology of LTs and their receptors, recent developments in the area of anti-LT strategies (in settings such as ongoing clinical studies), and prospects for future therapeutic applications.

## 1. Introduction

Lipid mediators, which denote bioactive mediators derived from lipids, play roles in immune regulation, self-defense, and the maintenance of homeostasis in living systems. They include prostaglandins (PGs) and leukotrienes (LTs), lysophospholipids (including sphingosine 1-phosphate), and endocannabinoids [1]. LTs, which are derived from arachidonic acid (5*Z*,8*Z*,11*Z*,14*Z*-eicosatetraenoic acid; AA) through two steps catalyzed by 5-lipoxygenase (5-LO), are inflammatory mediators that function in normal host defense and play roles in inflammatory diseases [2].

They exert their biological effects by binding to G-protein-coupled receptors (GPCRs). Different LT receptor subtypes exhibit unique functions and expression patterns. LT receptors BLT1 and BLT2 are activated by leukotriene B_4_ (LTB_4_), whereas CysLT1 and CysLT2 receptors are activated by cysteinyl LTs (CysLTs) [3]. LTs are involved in various inflammatory diseases, including asthma, allergic rhinitis (AR), atopic dermatitis (AD), allergic conjunctivitis, rheumatoid arthritis, anaphylaxis, chronic obstructive pulmonary disease (COPD), obliterative bronchiolitis after lung transplantation, and interstitial lung diseases [4]. This review summarizes the biology of LTs and their receptors, along with recent findings related to the pathophysiological roles of LTs and their signaling pathways in allergic diseases. We also discuss both current and future therapeutic prospects.

## 2. Biosynthesis and Metabolism of LTs

Lipids are the major components of cell membranes. Phospholipids containing AA, a polyunsaturated fatty acid comprising 20 carbon atoms, are an important component of cell membranes. In response to various stimuli, AA is released from phospholipids by phospholipase A_2_ (PLA_2_), particularly cytosolic PLA_2_α [1]. AA can be metabolized by various pathways, but two pathways, the 5-LO pathway and the cyclooxygenase (COX) pathway, are important in producing bioactive lipid mediators. The 5-LO pathway generates LTs from AA, while the COX pathway generates PGs and thromboxane [5,6].

**Figure 1:**
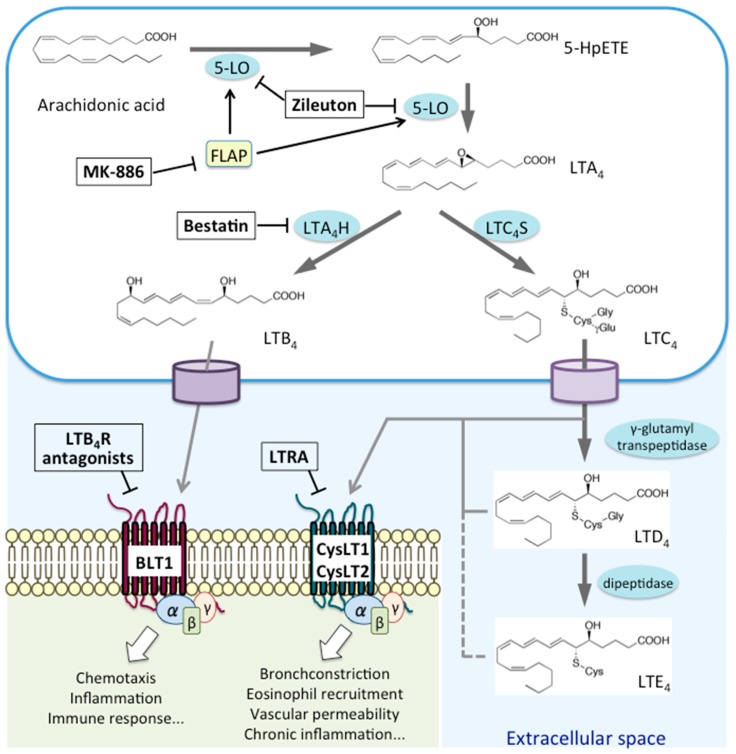
Biosynthetic pathways and receptors for leukotrienes.

LTs are classified into two groups: the chemoattractant LTB_4_, which only carries hydroxyl moieties, and CysLTs (LTC_4_, LTD_4_, and LTE_4_), which also carry amino acid moieties [7]. AA is first oxidized by 5-LO at the C-5 position to yield 5-hydroperoxyeicosatetraenoic acid (5-HpETE). It is then converted to an unstable intermediate, leukotriene A_4_ (LTA_4_); LTA_4_ is either converted to LTB_4_ by LTA_4_ hydrolase (LTA_4_H) or conjugated to reduced glutathione by leukotriene C_4_ synthase (LTC_4_S), yielding LTC_4_ [2]. LTC_4_ is then exported from the cell through transporters, such as multidrug-resistant protein 1, which recognize glutathione-containing molecules, and then converted to LTD_4_ and LTE_4_ by extracellular peptidases [7]. Signaling via BLT1 triggers chemotaxis, inflammation, and immune responses, whereas that through CysLTs results in bronchoconstriction, eosinophil recruitment, exacerbated vascular permeability, and chronic inflammation (Figure 1).

## 3. Expression of LT Receptors and Their Associated Signaling Pathways

Like other lipid mediators, PGs and LTs function as signaling molecules through specific GPCRs. BLT1 and BLT2 are activated by LTB_4_, whereas CysLT1 and CysLT2 are activated by CysLTs. There are many reports and controversies about the novel functions of LT-related signaling. Indeed, studies demonstrated BLT1 and BLT2 signaling in cancer. Whereas BLT1 was shown to accelerate tumor progression by enhancing inflammation [8], recent studies demonstrated that BLT1-mediated CD8^+^ T cell recruitment was essential for initiating antitumor immunity [9,10]. BLT2, on the other hand, accelerates chemotherapy resistance and tumor promotion [11]. The dual pro- and anti-inflammatory activities of the BLT2 receptor have added complexity, since BLT2-deficient mice exhibited reduced severity in arthritis models [12], whereas the deletion of BLT2 caused more severe colitis induced by dextran sulfate possibly due to the loss of BLT2-dependent intestinal barrier function [13]. Crosstalk between the CysLT1/2 and P2Y receptor systems has also been described [14,15].

Similar controversies surround novel receptors for CysLTs. For example, GPR99 was identified as a receptor for LTE_4_ [16]. The orphan GPR17, phylogenetically located at an intermediate position between the P2Y and CysLT receptors, was originally thought to be activated by CysLTs, nucleotides, and sugar nucleotides (UDP, UDP-glucose, and UDP-galactose) [17]; however, the action of GPR17 as a dual receptor for both CysLTs and nucleotides is yet to be proven [18]. Additionally, lipoxygenase metabolites derived from essential ω-3 polyunsaturated acids (the resolvins) activate the receptors GPR32 and ChemR23 [3]. The orphan receptor GPR32 was identified as a receptor for RvD1 [19], RvD5 [20], and RvD3 [21], and ChemR23 was initially described as one of three GPCRs that are activated by the chemotactic protein chemerin [22,23] (it is classified therefore as a proinflammatory receptor). However, subsequent studies using ChemR23 knockout mice as the basis for different disease models revealed its anti-inflammatory role. Indeed, ChemR23 was identified as a high-affinity RvE1 receptor through screening of the ability of RvE1 to inhibit TNFα-induced NF-κB activation in HEK293 cells transfected with candidate GPCRs [24].

### 3.1. LTB_4_ Receptors: BLT1 and BLT2

LTB_4_, which binds to the BLT1 receptor, is one of the most potent chemoattractants for many immune cells [25,26]. Studies report expression of BLT1 by nonmyeloid cells such as vascular smooth muscle cells, endothelial cells, skeletal muscle satellite cells, and neural stem cells, albeit at low levels [27,28,29]. Recent studies show that the LTB_4_-BLT1 axis plays a role in a variety of diseases other than allergic diseases; these include arthritis, COPD, cardiovascular diseases, age-related macular degeneration (AMD), cancer, and metabolic disorders (which are mentioned briefly in Section 6 below). 

The BLT2 gene lies adjacent to that of BLT1 in both humans and mice. BLT2 was first identified as a low-affinity receptor for LTB_4_ [30]; the dissociation constant (*K*d) (which is used to describe the affinity between a ligand and its cognate receptor) for binding of BLT2 to LTB_4_ is 20-fold higher than that of BLT1, and it induces calcium signaling at a 30-fold higher median effective concentration (EC_50_) (the concentration of ligand that induces a response halfway between the baseline and maximum) [31] than BLT1. Soon after, 12(*S*)-hydroxyheptadeca-5*Z*,8*E*,10*E*-trienoic acid (12-HHT), a downstream metabolite of COX enzymes, was identified as an endogenous ligand of BLT2 [32]. BLT2 is expressed at high levels by epithelial cells in various tissues, including intestine, skin, and cornea; the 12-HHT-BLT2 axis accelerates skin [33] and corneal [34] wound healing, and plays a protective role in acute lung injury [25,35,36].

### 3.2. CysLTs Receptors: CysLT1 and CysLT2

CysLTs, namely, LTC_4_, LTD_4_, and LTE_4_, are proinflammatory lipid mediators with bronchoconstricting ability. They are involved in the pathogenesis of allergic diseases such as asthma and AR. CysLTs are produced mainly by eosinophils, mast cells (MCs), and macrophages in response to a variety of stimuli [37]. CysLT1 is sensitive to classical CysLT antagonists such as montelukast, zafirlukast, pranlukast, pobilukast, and MK571. By contrast, CysLT2 is insensitive to these antagonists; indeed, there were no CysLT2-selective antagonists until recently [3,38]. Therefore, the role of the latter in allergic diseases remains unclear. Their affinity for CysLT1 is ranked as follows: LTD_4_ > LTC_4_ > LTE_4_ [39]. Meanwhile CysLT2 binds LTC_4_ and LTD_4_ with an affinity one log less than that of CysLT1 (binding rank order: LTD_4_ = LTC_4_ > LTE_4_) [40]. An ex vivo study of isolated human small bronchus revealed that the potency (as determined by their effect on bronchoconstriction) is LTC_4_ ≥ LTD_4_ > LTE_4_ [41].

CysLT1 is expressed by bronchial smooth muscle [39,42], whereas CysLT2 is expressed at high levels by endothelial cells within some vascular beds (including coronary vessels), and is implicated in a variety of cardiovascular functions such as increased vascular permeability and reduced systemic blood pressure [43].

### 3.3. GPR99

As stated above, both CysLT1 and CysLT2 have lower affinity to LTE_4_, the most stable CysLT. LTE_4_ is the most abundant CysLT present at sites of inflammation [44], where it induces airflow obstruction and eosinophil infiltration in patients with asthma. These facts suggest that another signaling pathway specific for LTE_4_ is involved in airway inflammation. GPR99, previously described as an oxoglutarate receptor (OXGR1), recently emerged as a potential novel receptor for LTE_4_ that mediates cutaneous vascular permeability in mice deficient in the terminal enzyme for CysLT biosynthesis, LTC_4_ synthase [16,45]. GPR99 is expressed on lung and nasal epithelial cells as well as on vascular smooth muscle cells in the nasal mucosa [46], where it modulates mucin release and goblet cell numbers [47]. Further studies are required to confirm whether GPR99 functions as a receptor for LTE_4_.

## 4. LTs and Allergic Diseases

### 4.1. Asthma

Asthma is a common and potentially serious chronic disease that imposes a substantial burden on patients. It causes respiratory symptoms, limits activity, and is prone to flare-ups (attacks) that sometimes require urgent healthcare and may be fatal. Asthma symptoms include wheezing, shortness of breath, chest tightness, and cough; the occurrence, frequency, and intensity of these symptoms vary over time. Difficulty in expelling air from the lungs is due to bronchoconstriction, airway wall thickening, and increased mucus production.

Some drugs, such as beta-blockers, aspirin, and NSAIDs, as well as viral infections, allergens, tobacco smoke, exercise, and stress, can trigger/induce asthma symptoms. The gold standard treatment is inhaled corticosteroid (ICS)-containing medication, but treatment should be customized to the individual patient (https://ginasthma.org/pocket-guide-for-asthma-management -and-prevention/).

#### 4.1.1. Pathology

Asthma is a complex and chronic disorder of the airways that is characterized by airflow obstruction, airway inflammation, and airway hyper-responsiveness (AHR). Airway inflammation plays an essential role in the pathogenesis of asthma and is characterized by infiltration of the airways by inflammatory cells such as neutrophils, eosinophils, and lymphocytes [48]. LT-mediated pathways are implicated in the pathophysiology of asthma [49]. The presence and elevated levels of numerous metabolites in the blood, sputum, and bronchoalveolar lavage (BAL) fluid from asthmatics or experimental animals supports this notion [49]. Asthma therapies that target CysLT1 are successful [50], but clinically are effective only in certain situations. Although one 5-LO inhibitor, zileuton, is approved for treatment of asthma [51], interference with other molecules in LT biosynthesis and signaling pathway means that it is not fully effective. Roles for BLT2 in asthma have been reported, although to a lesser extent than that of BLT1 [49,52,53,54].

#### 4.1.2. The LTB_4_–BLT1 Pathway in Asthma

The importance of the LTB_4_–BLT1 pathway in the pathogenesis of asthma is supported by extensive experimental data and findings (albeit limited) from clinical samples. For example, both levels of 5-LO and LTA_4_H in the airways and the number of circulating neutrophils are higher in patients with asthma than in those without [55,56]. Increased levels of LTB_4_ are detected in the blood, BAL fluid [57], and exhaled breath condensate (EBC) [58,59] from children and adolescents with asthma. In contrast to CysLTs, which are potent mediators of bronchoconstriction [48], LTB_4_ is a proinflammatory mediator that has a major effect on recruitment, activation, and survival of myeloid leukocytes [60]. Accumulation of neutrophils and eosinophils is considered to be a pathological parameter in asthma patients [61].

Based on the type of inflammatory cell in sputum, asthma is divided into four phenotypes: eosinophilic asthma, neutrophilic asthma, mixed granulocytic asthma, and paucigranulocytic asthma. Neutrophilic asthma is difficult to treat due to poor steroid responsiveness [62], being the predominant pattern in acute exacerbation of asthma in adults [63]. It occurs frequently in those with steroid-insensitive asthma [64,65,66], severe asthma [67], and occupational asthma [68].

Massive numbers of neutrophils are present in the airways of asthmatics suffering from clinical exacerbations or asthma-related sudden death [69,70]. Therefore, targeting and modulating the LTB_4_–BLT1 pathway will offer innovative therapeutic opportunities, especially for patients with asthma that remains uncontrolled despite intensive corticosteroid treatment [49].

In addition to its important role as a neutrophil chemoattractant, another important role of the LTB_4_–BLT1 pathway (despite observations made more than two decades ago) was shown only recently; the pathway plays an important role in recruiting and activating T cells [71] and dendritic cells, the major antigen-presenting cell in the lung [49]. Allergen-specific memory T cells and IgE-specific antibodies are required for these responses. It is now recognized that many T cell subtypes, such as CD4^+^ and CD8^+^ T cells, contribute to the pathophysiology and development of allergic airway responses [72]. BLT1-deficient mice are resistant to OVA-induced allergic AHR and show reduced accumulation of neutrophils, eosinophils [73], lymphocytes [74], and dendritic cells in the lungs [75]. In agreement with a mouse model of allergic pulmonary inflammation [76], large numbers of BLT1-expressing effector memory CD8^+^ T cells are present in BAL fluid from asthmatic patients [77].

Although accumulating evidence demonstrates the importance of LTB_4_ in asthma, previous studies failed to show the effectiveness of targeting the LTB_4_–BLT1 pathway as a treatment for asthma. A clinical trial of the BLT receptor antagonist LY293111 in 12 asthmatic patients showed that it led to a significant reduction in the number of neutrophils in BAL fluid, but failed to improve respiratory function or airway reactivity after allergen challenge [78]. Studies in a guinea pig asthma model showed that LY293111 had no effect on eosinophil and macrophage infiltration into the BAL fluid [79]. However, a study examining the role of another BLT1 antagonist, CP-105,696, in monkeys showed that the compound inhibited LTB_4_-mediated neutrophil chemotaxis and upregulation of CD11b^+^ cells in BAL, leading to amelioration of AHR [80].

A recent in vivo study in BLT2-deficient mice indicates that BLT2 protects against allergic airway inflammation and that reduced expression of BLT2 by CD4^+^ T cells might contribute to the pathophysiology of asthma [52]. Also, an increasing number of studies have demonstrated that BLT2 has a protective role in allergic airway inflammation [49,53,54].

#### 4.1.3. The CysLT Pathway in Asthma

CysLTs are thought to be essential for the pathogenesis of acute and chronic asthma because they are the most potent bronchoconstrictors in humans [48,81]; indeed, they are much more potent than histamine [82]. CysLTs are detected in blood, BAL fluid, and urine samples from asthmatic patients after bronchospasm. The contribution of the CysLT1 signaling pathway in the clinical manifestations of asthma is supported by the finding that, in addition to the 5-LO inhibitor zileuton [51], CysLT receptor antagonists or leukotriene receptor antagonists (LTRAs) montelukast, zafirlukast, and pranlukast are effective treatments [37,83]. Moreover, epigenetic changes in the genes encoding CysLT1 and LTC_4_S confer susceptibility to asthma. Particulate matter with an aerodynamic diameter of <2.5 μm (PM2.5) exacerbates asthma symptoms [84], and a recent genome-wide study demonstrated that the effects of acute PM2.5 exposure are associated with changes in CysLT1 expression and methylation of CpG sites on the CysLT1 and LTC_4_S genes [85].

In recent years, accumulating evidence has shown that CysLTs, among other mediators (cytokines, chemokines, growth factors, alarmins, and lipid mediators), play essential roles in regulating eosinophil recruitment [37]. Indeed, CysLTs display eosinophilotactic activity in vitro via CysLT1 [86,87]. In vivo, involvement of CysLTs in eosinophil influx was first demonstrated in guinea pigs in the 1990s [88], and then in humans [89]. Recently, a study described the roles of LTC_4_ in mediating eosinophil trafficking from the lungs to the paratracheal lymph nodes in experimental allergic asthma [90]. Activation of CysLT1 is also important for eosinophilic inflammation because it upregulates expression of endothelial adhesion molecules [86,91], induces eosinophil chemotaxis, and reduces eosinophil apoptosis [92]. Eosinophils are thought to be the main cellular source of CysLTs; these cells express CysLT1 on the membrane, suggesting that this molecule has autocrine activity [93].

Studies show that respiratory parameters related to lung microvascular leakage and lung edema in antigen-challenged Brown Norway rats are improved partially by inhibitors of AA metabolism; these inhibitors include indomethacin (a COX inhibitor) and montelukast. These data indicate that one of the mechanisms by which CysLTs affect the pathophysiology of asthma is based on CysLT-dependent exacerbation of vascular leakage and mucosal edema [94].

CysLTs also play a role in airway remodeling by promoting proliferation of airway smooth muscle cells [95] and epithelial cells [96], and by increasing collagen deposition [97], which is an important feature of chronic asthma [98].

It is not clear how CysLT2 signaling contributes to the pathophysiology and manifestations of asthma since no specific antagonists were available until recently [37]. Currently, an increasing number of reports demonstrate involvement of CysLT2 [91,99]. Future studies will likely support a role for CysLT2 in the pathophysiology of asthma.

### 4.2. Exercise-Induced Asthma (EIA)

EIA, a type of asthma common in children, causes airway narrowing during exercise; symptoms include cough and shortness of breath [100]. Studies report involvement of CysLTs in EIA [101]. In addition, leukotriene receptor antagonists (LTRAs) and ICS are useful for long-term management of asthma patients complicated by EIA and AR [102].

Several observational studies suggest involvement of LTs in EIA; one study reports that exercise-induced stress increases transcription of genes encoding 5-LO and 5-LO-activating protein (FLAP), thereby increasing production of LTB_4_ and LTC_4_ in plasma after exercise [103], whereas another reports that levels of CysLTs in EBC from EIA patients are higher than those in controls, and that they increase after exercise challenge [104]. Despite the clinical effectiveness of LTRAs on EIA, few experimental studies have examined the role of LTs in the pathogenesis of EIA. One study that analyzed LTB_4_ production by peripheral blood mononuclear cells (PBMCs) isolated before and after EIA demonstrated a several-fold increase in production of LTB_4_ by PBMCs isolated after EIA when compared with those isolated before exercise [105].

### 4.3. Aspirin-Sensitive Asthma (ASA)

ASA is a particular phenotype of severe late-onset asthma. The correlation between aspirin sensitivity, asthma, and nasal polyposis was recognized in the early 20th century. Today, chronic rhinosinusitis with nasal polyposis, bronchial asthma, and reactions to aspirin or COX-1 inhibitors (eponymously named Samter’s Triad) is a manifestation of aspirin-exacerbated respiratory disease (AERD) [106]. AERD affects approximately 0.3–0.9% of the general population in the USA, and approximately 7% of those with asthma [107].

The pathophysiology of AERD is characterized by a non-IgE hypersensitivity reaction to ASA/COX-1 inhibitors; the reaction is commonly comorbid and not due to underlying allergic diseases [6]. AERD is thought to be caused by abnormalities in AA biosynthesis [108,109]. Importantly, specific COX-2 inhibitors do not cause respiratory reactions in patients with AERD [110].

The mechanism underlying AERD is likely to relate to constitutive overproduction of CysLTs, with a concomitant decrease in downstream products of the COX-1 pathway, the latter of which have an inherent inhibitory effect on CysLTs [109,111]. This results in a proinflammatory environment. Indeed, CysLTs are implicated in development of rhinitis and AERD through three mechanisms: (1) increased vasodilation and permeability of the nasal vasculature leading to mucosal edema and nasal congestion; (2) increased inflammation at the level of the sinonasal epithelium resulting in more mucus production and rhinorrhea; and (3) augmented inflammation through recruitment of inflammatory cells [112]. The elevated levels of CysLTs found in the urine, sputum, exhaled breath, and peripheral blood of AERD patients support this theory [108,112].

Investigations into the precise mechanisms underlying the constitutive overproduction of CysLTs are ongoing. One study examined the possible mechanism underlying increased levels of CysLTs in AERD. The report showed that the cytokine milieu in AERD did contain higher levels of IFN-γ than that in those with asthmatic or eosinophilic sinusitis; increased IFN-γ stimulated differentiation of eosinophils, leading to marked upregulation of the number of infiltrating eosinophils; in turn, those IFN-γ-differentiated eosinophils increased the levels of LTC_4_ [113]. Moreover, eosinophils secrete numerous cytokines and chemokines such as interleukin (IL)-4. Both IL-4 and IFN-γ upregulate expression of CysLT1 by multiple cell lines, including eosinophils and MCs [114]. According to several studies that examined the role of IL-33 in AERD, IL-33 may act as a bridge between CysLT overexpression and MC activation, which is one of the characteristics of AERD. CysLTs induce IL-33 expression, and IL-33 stimulates MCs to generate PGD_2_, thromboxane A_2_, and CysLTs [115]. In addition, platelet-adherent leukocytes are effectors of AERD and lead to increased levels of CysLTs [116]. Finally, lipoxins are anti-inflammatory mediators that typically downregulate expression of proinflammatory cytokines by competing for the CysLT1 receptor. Interestingly, although CysLT1 is upregulated in patients with AERD, there is a simultaneous downregulation of lipoxins, leading to inadequate competition with CysLTs for the receptor; this contributes to CysLT-driven pathophysiology [116,117,118].

Finally, another study demonstrated possible involvement of CysLT2 in ASA; CysLT2 signaling, IL-33-dependent ILC2 (innate lymphoid cells) expansion, and IL-33-driven MC activation are necessary for induction of type 2 immunopathology and aspirin sensitivity, indicating that CysLT2 might be a potential target for the treatment of ASA [119].

### 4.4. Allergic Rhinitis (AR)

AR, which is characterized clinically by sneezing, rhinorrhea, nasal itching, and congestion, is an allergen-driven mucosal inflammatory disease that is modulated by IgE. Epidemiological studies indicate that the prevalence of AR continues to increase; indeed, it is a worldwide health problem that places a significant healthcare burden on individuals and society [120]. AR affects 20–30% of adults both in the United States and Europe, and the prevalence is perhaps somewhat higher in children [121].

CysLTs increase vascular permeability, leading to nasal congestion, increased mucus production and secretion, rhinorrhea, and recruitment of inflammatory cells into tissues. Recent evidence suggests involvement of CysLTs in the pathophysiology of AR: CysLTs are released from inflammatory cells that participate in AR [122], receptors for CysLTs are located in nasal tissue [123], and CysLT levels are increased in nasal lavage fluid from patients with AR [124]. Increasing numbers of studies demonstrate that patients with AR respond favorably to treatment with CysLT receptor antagonists. By binding competitively to CysLT1, LTRAs such as montelukast, zafirlukast, and pranlukast block the effects of CysLTs and improve the symptoms of chronic respiratory diseases, particularly bronchial asthma and AR [120]. A recent meta-analysis suggests that H1-antihistamines (SAH) and LTRA have similar effects and safety profiles when used to treat seasonal AR; however, SAH is more appropriate for daytime nasal symptoms (congestion, rhinorrhea, pruritus, and sneezing) while LTRA is better suited for night-time symptoms (difficulty going to sleep, night-time awakenings, and nasal congestion on waking) [125]. Accumulating evidence shows the effectiveness of combination therapy with LTRAs and AH. One meta-analysis shows that, compared with SAH alone, LTRAs + SAH show increased therapeutic efficacy against daytime and composite nasal symptoms, although they do not affect night-time nasal symptoms and eye symptoms. The study also demonstrates the possibility that patients with perennial AR benefit more from combination therapy [126]. Another study also shows that combination therapy with LTRA + SAH provides greater beneficial effects against composite nasal symptoms, rhinorrhea, sneezing, and rhinoconjunctivitis symptoms in patients with AR and patients with perennial AR [127].

The role of LTB_4_ in AR is unclear, although several studies report that patients with AR show increased levels of LTB_4_ in the nasal cavity and EBC [128], suggesting involvement of LTB_4_ in the pathogenesis of AR.

Overall, antileukotrienes, in combination with SAH, are effective against AR and result in significant improvements in daytime nasal symptoms and quality of life.

### 4.5. Atopic Dermatitis (AD)

AD is the most common inflammatory dermatological disorder. It is either chronic or chronically relapsing, and characterized by intense pruritus and dry skin. The incidence of AD has increased over the last few decades; indeed, 10–25% of children and 2–8% of adults in affluent nations are affected. Of all patients affected, up to one quarter have moderate-to-severe disease [129]. Approximately one-third of patients carry the disease into adulthood; thus the disease becomes life-long. Glucocorticoids have long been the gold standard treatment, but use is limited by their adverse side effects [130].

The underlying etiopathogenesis of AD is multifaceted; however, key elements are an impaired skin barrier, dysregulated immune responses, immunologic abnormalities, and subsequent release of inflammatory mediators [131]. The role of LTs and PGs in development of AD was established prototypically in studies of asthma pathogenesis. As in asthma, high concentrations of eicosanoids are present in skin affected by AD. PGD_2_ is the main PG produced by MCs [132]; PGE_2_ and LTB_4_ are also present at high concentrations [133]. Additionally, a recent study that investigated serum metabolic abnormalities in AD children found that the differential metabolites, including LTB_4_ and PGs, were associated with inflammatory responses and bile acid metabolism [134]. An animal study based on NC/Nga mice demonstrated that treatment with docosahexaenoic acid (DHA)/eicosapentaenoic acid (EPA) and FK506 reduced the clinical scores for dermatitis and lowered local LTB_4_ concentrations, suggesting that DHA/EPA might be effective against skin inflammation in AD by suppressing local LTB_4_ production [135].

Recent studies show that LTB_4_ and CysLTs play important but distinct roles in the pathogenesis of AD. LTB_4_ is implicated in recruitment of neutrophils and Th2 cells, and it is thought to be pivotal for the pathogenesis of acute inflammation in both human and murine models of AD [136,137]. CysLTs, on the other hand, are implicated in chronic inflammatory characteristics of AD, including collagen deposition, skin thickening, and fibrosis, all of which are typical of chronic AD [138]. Thus, it follows that therapies targeting LTB_4_ or CysLTs might be promising for management of AD. However, montelukast has questionable efficacy in patients with AD; randomized controlled trials demonstrate limited and contradictory evidence regarding the efficacy of montelukast compared with placebo, with larger trials suggesting no clinically significant effect [139,140,141]. Meanwhile, small pilot studies using oral zileuton show some potential efficacy in humans and canines [142,143]. Since these studies were limited in size, they should be interpreted with caution. Larger-scaled clinical research is needed before a definitive conclusion can be drawn. However, development of eicosanoid-directed therapies specifically targeting the pathophysiology underlying AD will be important.

Several agents that target LTs and/or PGs are in development. These include OC000459, Q301, and ZPL-521. Timapiprant (OC000459), a novel oral antagonist for chemoattractant receptor-homologous molecules expressed by Th2 cells (CRTH2), has proven to be safe and effective for management of asthma [144]; however, it failed to show efficacy against AD in clinical trials (https://clinicaltrials.gov/ct2/show/NCT02002208). Q301 (Qurient, Gyoenggi-do, Republic of Korea) is a novel topical zileuton cream [Nam, KY, Kim, JJ, OH, SH, et al. (2016). Topical anti-inflammatory pharmaceutical composition with zileuton cream formulation. US patent application 20160228353]. Although clinical trials failed to show significant efficacy of montelukast against AD, intervention in the LTB_4_–BLT1 pathway via 5-LO inhibition might be effective since LTB_4_ seems to be the most potent LT with respect to triggering the proinflammatory cascade and recruitment of Th2 cells; by contrast, CysLTs are involved in the chronic inflammatory course of AD [136,137,138]. A phase 2 clinical trial measuring safety and efficacy of Q301 with respect to moderate-to-severe AD (NCT02426359) was completed recently (https://clinicaltrials.gov/ct2/show/NCT02426359). It remains unclear whether anti-LT therapy using oral zileuton or topical Q301 will be clinically significant, but these therapeutic options might provide some symptomatic relief.

A novel cPLA_2_ inhibitor reduces allergic inflammation in an in vitro model of asthma [145]. ZPL-521 (Ziarco, Canterbury, UK), a topical formulation of this cPLA_2_ inhibitor, has completed phase 1 and 2 clinical trials (NCT02795832) to evaluate its safety and efficacy when used to treat moderate-to-severe AD (https://clinicaltrials.gov/ct2/show/NCT02795832). The results remain to be seen. The interactions between LTs and PGs during the pathogenesis of AD are complex, and broad downregulation of the AA pathway would certainly lead to a reduction in levels of proinflammatory LTs and PGs; however, this approach also inhibits regulatory interactions, such as PGD_2_–DP1 signaling.

Overall, eicosanoids such as LTs and PGs are involved in a number of complex pathophysiologic changes seen in the skin of patients with AD. Medications targeting LTs will provide a therapeutic avenue for patients suffering from moderate-to-severe AD. Several medications are in the developmental pipeline, although their efficacy remains to be determined; these topical agents may become effective maintenance treatments or flare prophylactics for those with AD (similar to antileukotrienes for asthma) [146].

### 4.6. Allergic Conjunctivitis

Ocular itching, lacrimation, and redness are common symptoms of allergic conjunctivitis, which is usually considered a comorbidity associated with AR. Ocular allergy occurs through activation of Th2 cell-mediated cascades, leading to production of IgE; it can also occur in combination with T lymphocyte-mediated disorders, with subsequent development of acute and chronic forms of ocular allergy. This proinflammatory state caused by activation of transcription factors creates an immune cascade via increased cellular infiltration (e.g., eosinophils) and secretion of chemokines, cytokines, and metalloproteinases, which further promotes ocular surface damage and disruption of epithelial barriers [147].

LTB_4_ was first shown to cause eosinophil migration into conjunctival tissue in a guinea pig model. Combination therapy with an SAH and a 5-LO inhibitor results in near-complete suppression of allergic conjunctivitis in this model [148]. Involvement of the LTB_4_–BLT1 pathway in the pathophysiology of allergic conjunctivitis was further demonstrated in a mouse model of ocular allergy in which the BLT1 antagonist ONO-4057 inhibited ocular scratching behavior induced by challenge with ragweed pollen [149]. LTB_4_ is also involved in secondary allergic conjunctivitis; indeed, a recent study investigating changes in the concentration of inflammatory mediators (including histamine and LTs) in tears of those with secondary allergic conjunctivitis due to primary allergic reactions in the nasal mucosa revealed involvement of LTB_4_ in both late and delayed-type secondary allergic conjunctivitis [150].

One of the main mechanisms underlying the pathogenesis of allergic conjunctivitis is increased secretion of mucin by goblet cells. Both CysLT1 and CysLT2 are expressed by rat conjunctiva and in cultured rat and human conjunctival goblet cells. Treatment with the CysLT1 antagonist MK571 led to a marked reduction in the amount of mucin secreted by LTD_4_-stimulated goblet cells [151]. Involvement of the CysLTs pathway in allergic conjunctivitis was also indicated in a clinical trial of orally administered montelukast in patients with both vernal keratoconjunctivitis and asthma; the trial reported a reduction in the severity of ocular signs and symptoms [152].

There are limited approved therapeutic options for treatment of allergic conjunctivitis. Of interest, it was confirmed that the nonpharmacological interventions of artificial tears and the use of cold compresses alone or in combination often provide patients with significant benefits [147]. In the area of pharmacotherapy, there has been one new approval of an antihistamine, cetirizine. Considering that the LTB_4_–BLT1 pathway is involved in the pathophysiology of allergic conjunctivitis, the therapeutic use of BLT1 antagonists in addition to antihistamines may offer more effective treatment.

### 4.7. Anaphylaxis

Anaphylaxis is a life-threatening allergic reaction in which an antigen binds to IgE and activates MCs and basophils, leading to massive release of inflammatory mediators [153]. The resulting increase in vascular permeability exacerbates the symptoms. Increased LTE_4_ levels are associated with anaphylaxis [154]. MCs are thought to be the main source of CysLTs during anaphylactic reactions [155,156].

An animal study demonstrated reduced extravasation of plasma proteins in CysLT1-deficient mice undergoing IgE-mediated passive cutaneous anaphylaxis [155]. By contrast, CysLT2 transgenic mice showed increased vascular permeability in response to endogenous CysLTs produced by activated MCs during passive cutaneous anaphylaxis [43]. Both of these animal studies suggest that the CysLTs pathway, at least under certain conditions, regulates endothelial integrity.

MC activation disorders include mastocytosis (a spectrum of rare and well-defined diseases associated with clonal expansion of MCs in the skin and/or other tissues and organs) and MC activation syndrome (MCAS) (a rare condition defined by acute and/or chronic symptoms of MC activation with elevation of MC mediators at baseline or during acute episodes, without MC hyperplasia) [157]. Primary MC activation disorders are caused by intrinsic proliferation or clonal expansion of MCs and are associated typically with KIT mutations, represented by cutaneous or systemic mastocytosis (CM or SM) and monoclonal MCAS [158]. Secondary MC disorders are associated with allergic and other inflammatory or autoimmune disorders.

Symptoms associated with MC activation arise secondary to tissue responses to inflammatory mediators such as tryptase, histamine, PGs, LTs, and cytokines as well as the local MC burden in patients with mastocytosis [159]. Symptoms can be managed by blocking mediator receptors (H1- and H2-antihistamines and LT receptors), inhibiting mediator synthesis (aspirin and zileuton) and mediator release (sodium cromolyn), or instigating anti-IgE therapy, or a combination of these.

As stated below, measurement of urinary levels of excreted LTE_4_, the stable CysLT derivative of LTC_4_, can be used to quantitate whole-body production of CysLTs, which reflects short-term changes in secretion [154]. MCs, basophils, and eosinophils synthesize LTs, which are also produced by platelets and endothelial cells via transcellular biosynthesis [160]. A 5.5- to 52-fold increase in urinary CysLT has been reported during anaphylactic reactions [161]. More prominent elevation of urinary LTE_4_ levels was observed among patients with SM, than among nonmastocytosis populations with symptoms ascribed to excessive release of MC mediators (e.g., chronic urticaria, idiopathic angioedema, and drug allergy) [154].

Pathophysiological roles of leukotrienes and antileukotriene strategy for allergic diseases in clinical and experimental settings are described in Table 1.

## 5. Urinary LTE_4_ as a Biomarker of Allergic Disease

Measurement of urinary LTE_4_ (uLTE_4_), the stable CysLT derivative of LTC_4_, can be a useful noninvasive method for measuring whole-body CysLT levels; indeed, it is a biomarker of total production and excretion of CysLTs. The level of LTE_4_ in serum is too low to measure. Studies show that inhalation of LTC_4_ or LTD_4_ leads to a dose-dependent increase in uLTE_4_ levels [162]. Approximately 5% of inhaled CysLTs were excreted in urine [163], almost all in the form of uLTE_4_ [47].

Mass spectrometry is the gold standard for measurement of uLTE_4_ because it has a low coefficient of variation and almost 100% recovery from spiked samples, indicating high sensitivity and precision [164,165]. The measurement is expressed in terms of picograms of LTE_4_ per milligram of creatinine (Cr); the reference range among normal volunteers is <104 pg/mg Cr [165]. Increased urinary levels of LTE_4_ are reported in several conditions, including AERD [166], in which MC activation occurs.

Overall, measurement of uLTE_4_ is a sensitive and noninvasive method of assaying total body production of CysLTs and is a useful biomarker of exposure to atopic and nonatopic asthma triggers [47,167], recent asthma exacerbations [168], AERD [166,169], early development of childhood atopy [170,171], and obstructive sleep apnea [172,173]; it is also useful for predicting susceptibility to LT receptor antagonists [174,175,176].

## 6. Other Diseases

**Figure 2:**
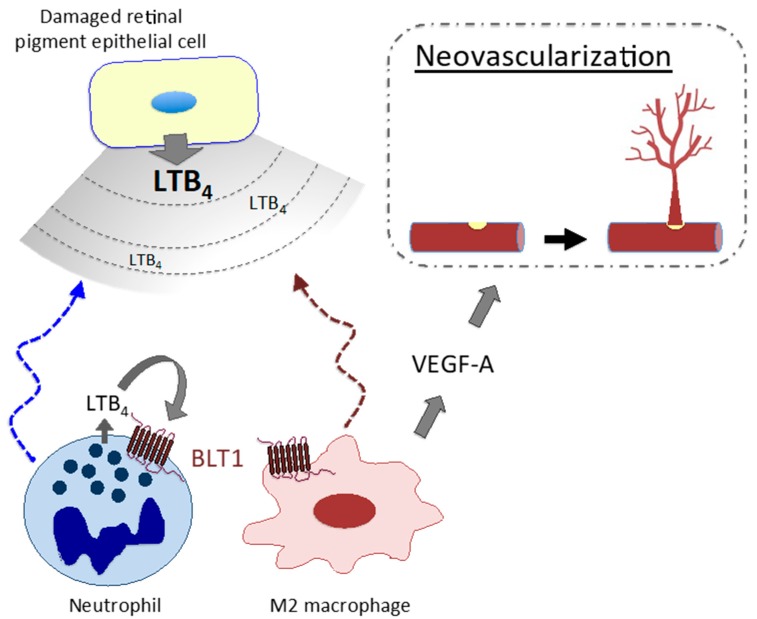
M2 macrophages promote abnormal (pathogenic) neovascularization in age-related macular degeneration via the LTB_4_–BLT1 signaling pathway.

LT signaling is involved in the pathogenesis/pathophysiology of various diseases other than allergic diseases. Studies demonstrate involvement of LTB_4_–BLT1 signaling in the pathogenesis/pathophysiology of endotoxin shock [177], rheumatoid arthritis [178], inflammatory colitis [13,179], alveolar bone loss [180], osteoporosis [181], epidermolysis bullosa acquisita [182], contact dermatitis [183,184], psoriasis [185], and silica-induced lung cancer [8,186]. In addition, our recent study revealed that M2 macrophages expressing BLT1 augment neovascularization in a mouse model of AMD, indicating involvement of LTB_4_–BLT1 signaling in disease pathogenesis and strongly suggesting a potential novel therapeutic target for neovascularization [187]. Damaged retinal pigment epithelial cells release LTB_4_, which recruits immune cells such as neutrophils and inflammatory monocytes/macrophages to the injured retina. An autocrine/paracrine loop of LTB_4_ production in these cells leads to formation of an inflammatory environment. Among these cells, M2 macrophages secrete VEGF-A, which accelerates choroidal neovascularization (Figure 2).

## 7. Conclusions

In this review, we first summarized the biosynthetic and metabolic pathways of LTs and described expression and signaling pathways of leukotriene receptors (Figure 1). Next, we discussed the pathophysiological roles of LTs in allergic diseases and provided up-to-date information obtained from both experimental and clinical settings. Meanwhile antileukotriene treatment has become standard therapy for some allergic diseases, several clinical trials are ongoing, and animal studies suggest further applications for anti-LT strategies in many allergic conditions. Because LTs play a major role in the pathophysiology of acute and chronic inflammation (i.e., neutrophil recruitment and airway remodeling), targeting their signaling pathways should be a promising therapeutic option for various allergic diseases.

## Figures and Tables

**Table 1:** Pathophysiological roles of leukotrienes and treatment strategies for allergic diseases.

Disease	Roles of the LTB_4_-BLT1 Pathway	Roles of CysLT Pathways	Anti-leukotriene Standard Therapy in a Clinical Trial *Experimentally Effective*
Asthma	Th2-type immune response Recruitment of granulocytes Recruitment of effector CD8^+^ T cells	Bronchoconstriction Eosinophil recruitment Airway remodeling (proliferation of airway smooth muscle cells and epithelial cells; deposition of collagen)	**Leukotriene receptor antagonist (LTRA)** **5-LO inhibitor (zileuton)**
Neutrophilic asthma	Neutrophil chemotaxis Recruitment and activation of allergen-specific memory T cells		*BLT1 antagonist (LY293111, CP-105,696)*
Aspirin-exacerbated respiratory disease; aspirin-sensitive asthma		Increased vasodilation Nasal vasculature permeability Mucus production Recruitment of inflammatory cells Eosinophil infiltration	
Allergic rhinitis		Enhanced vascular permeability Recruitment of inflammatory cells	**LTRA**
Atopic dermatitis	Acute inflammation: recruitment of neutrophils and Th2 cells	Chronic inflammatory state: collagen deposition, skin thickening, and fibrosis	Q301 (zileuton cream)? ZPL-521 (cPLA_2_ inhibitor ointment)? *5-LO inhibitor (zileuton)*
Allergic conjunctivitis	Eosinophil migration	Increased secretion of mucin by goblet cells	LTRA (montelukast) *BLT1 antagonist (ONO-4057)* *5-LO inhibitor (zileuton)*

## Abbreviations

PG	prostaglandin
LT	leukotriene
AA	arachidonic acid
GPCR	G-protein-coupled receptor
LTB_4_	leukotriene B_4_
PLA_2_	phospholipase A_2_
5-LO	5-lipoxygenase
COX	cyclooxygenase
5-HpETE	5-hydroxyperoxyeicosatetraenoic acid
LTA_4_	leukotriene A_4_
LTA_4_H	leukotriene A_4_ hydrolase
LTC_4_S	leukotriene C_4_ synthase
COPD	chronic obstructive pulmonary disease
AMD	age-related macular degeneration
BLT1	LTB_4_ receptor 1
BLT2	LTB_4_ receptor 2
12-HHT	12(*S*)-hydroxyheptadeca-5*Z*,8*E*,10*E*-trienoic acid
OXGR1	oxoglutarate receptor
ICS	inhaled corticosteroid
AHR	hyper-responsiveness
BAL	bronchoalveolar lavage
EIA	Exercise-induced asthma
LTRA	leukotriene receptor antagonist
FLAP	five-lipoxygenase-activating protein
EBC	exhaled breath condensate
ASA	aspirin-sensitive asthma
AERD	aspirin-exacerbated respiratory disease
IL	interleukin
AR	allergic rhinitis
SAH	H1-antihistamines
AD	atopic dermatitis
MC	mast cell
MCAS	mast cell activation syndrome
SM	systemic mastocytosis
uLTE_4_	urinary LTE_4_
